# Hantavirus infection-related acute inflammatory demyelinative polyradiculoneuropathy: A case report and literature review

**DOI:** 10.1097/MD.0000000000037332

**Published:** 2024-03-08

**Authors:** Ying Zhou, Jun Yang, Hong Hai, Jun Dong, Ying Wen

**Affiliations:** a Department of Infectious Diseases II, Shenyang Natural Focal Diseases Clinical Medical Research Center, The First Affiliated Hospital of China Medical University. No. 155, Nanjing North Street, Heping District, Shenyang 110001, Liaoning Province, China; b Department of neurology, The First Affiliated Hospital of China Medical University. No. 155, Nanjing North Street, Heping District, Shenyang 110001, Liaoning Province, China; c Department of rehabilitation, The First Affiliated Hospital of China Medical University, No. 155, Nanjing North Street, Heping District, Shenyang, Liaoning Province, China; d Department of Infectious Diseases Ⅲ, Fushun Infectious Disease Hospital, No. 25 Shenfu North Line, Shuncheng District, Fushun City, Liaoning Province, China

**Keywords:** a multidisciplinary team, clinical features, Guillain–Barre syndrome, hantavirus, hemorrhagic fever with renal syndrome

## Abstract

**Rationale:**

Hemorrhagic fever with renal syndrome (HFRS) is a common infectious disease in China. As a complication of post-Hantavirus infection, Guillain–Barre syndrome (GBS) was rarely previously reported. Here, we described a case of acute inflammatory demyelinative polyradiculoneuropathy secondary to Hantavirus infection in spring of 2023. We also made a summary of the clinical features from previous reported cases.

**Patient concerns:**

A young male patient complained a fever with headache, who was subsequently diagnosed with HFRS with positive serum Hantavirus antibody IgM. Two weeks later, he presented sustained back pain, obvious numbness located in 4 extremities, chest and abdomen, facial dyskinesia and 4 extremities muscle weakness.

**Diagnosis, Interventions, and Outcomes:**

He was rapidly diagnosed with GBS by typical cerebrospinal fluid change and the electromyography examination presentation, which was verified associated with hantavirus infection. He was treated with intravenous immunoglobulin infusion followed by rehabilitation treatment. He got a complete recovery within 4 months after disease onset.

**Lessons:**

GBS was an uncommon manifestation of Hantavirus infection. GBS should be considered when acute limb weakness happens in cases with HFRS. A multidisciplinary team could make a rapid diagnosis and optimal treatment when nervous system disorders occurred.

## 1. Introduction

Fever, thrombocytopenia, acute kidney injury and proteinuria are the characters of hemorrhagic fever with renal syndrome (HFRS). Hantavirus infection was also involved in central/peripheral nervous system disorder, including encephalitis^[1–5]^ and pituitary glands injury/hypopituitarism,^[6–15]^ Guillain–Barre syndrome (GBS)^[9,16–19]^ and acute-onset chronic inflammatory demyelinating polyneuropathy.^[20]^ Imaging is nonspecific but helpful for diagnosis and differential diagnosis.^[21]^ Here, we reported a case of mild HFRS, who developed subsequent acute inflammatory demyelinating polyradiculoneuropathy (AIDP), and experienced clinical improvement after intravenous immunoglobulins (IVIG) followed by rehabilitation treatment.

## 2. Case presentation

**Figure 1.**
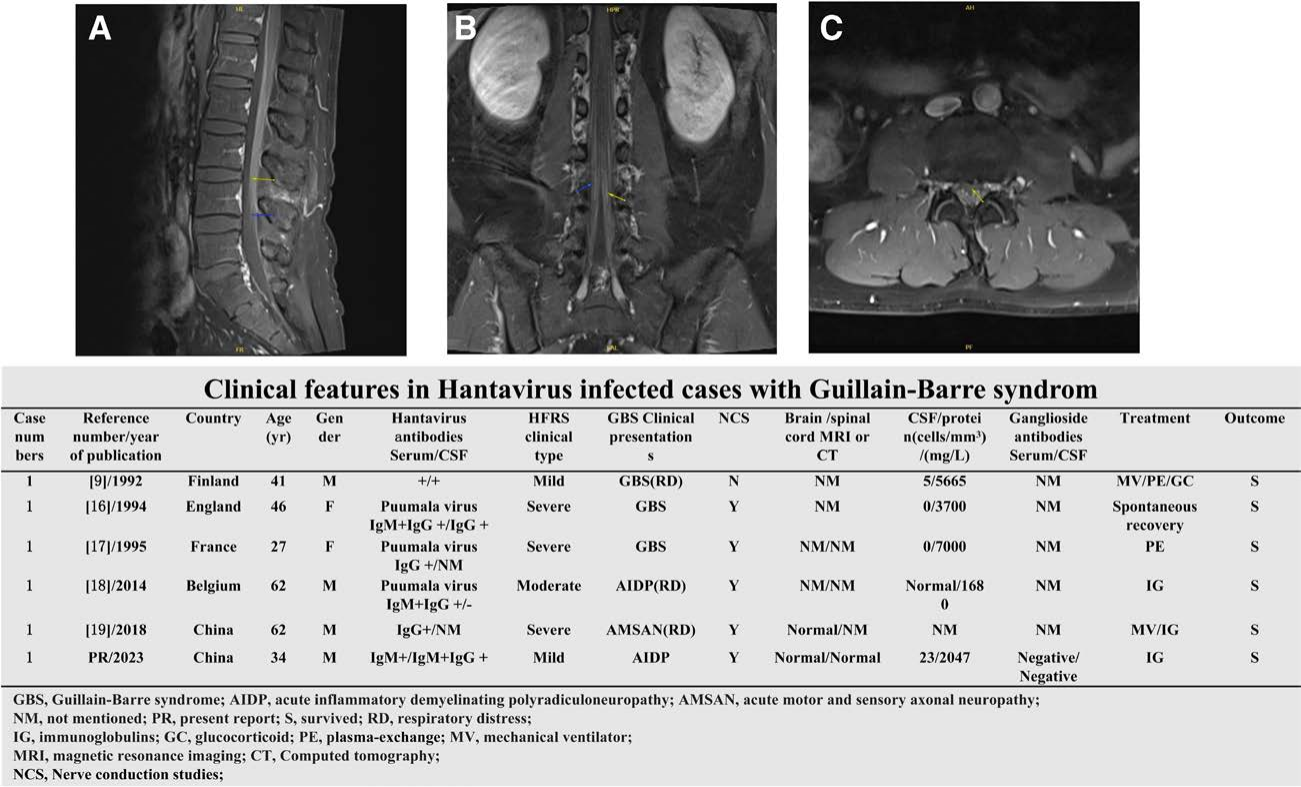
Gadolinium-enhanced MRI of lumbar segment of spinal cord of this case and clinical features in Hantavirus infected cases with Guillain–Barre syndrome. The arrows showed the linear enhancement at the nerve root—(A) sagitta, (B) coronal, and (C) transverse view.

A 34-year-old Chinese man living in a county town of Liaoning province in northeast China, complained a fever with headache in April 2, 2023, was admitted to local hospital. Abnormal laboratory results included elevated peripheral blood white cell counts (10.45 × 10^9^/L), decreased platelet counts (76 × 10^9^/L), increased serum creatinine level (119 μmol/L) and positive urine protein (++), positive serum Hantavirus antibody IgM. Although his urine volume was normal, the ultrasound examination showed the coarse and slightly enhanced echo of bilateral kidneys parenchyma. The HFRS was considered. On April 8, 2023, his temperature was normal and peripheral blood white blood cell counts, platelet counts, and serum creatinine level returned to normal. On April 12, 2023, he complained sustained back pain, followed by obvious numbness located in 4 extremities, chest and abdomen, accompanied by facial dyskinesia and 4 extremities muscle weakness. Then he adopted a referral to our hospital in April 18, 2023. The physical examination presented dysfunction in right eyes closing, frowning, grinning, and cheek blowing. The weakened muscle power was observed in double upper limb (grade 5-/5) and double lower limb (grade 4-/5). The decreased muscle tone of 4 extremities and decreased deep tendon reflexes were also found without pathological reflex. He presented mild difficulty of urination and defecation. He had no arrhythmia and blood pressure fluctuation. He had no respiratory distress and dysphagia. Brain magnetic resonance imaging (MRI) was normal, and spinal gadolinium-enhanced MRI showed linear enhancement at the nerve root (Fig. 1). The cerebrospinal fluid (CSF) pressure was 150 mm H_2_O, protein level was 2047 mg/L, cell count was 23 × 10^6^/L, acid-fast stain and India ink stain were negative, Hantavirus antibody IgM and IgG (Diagnostic kit for antibody to Hantavirus-colloidal gold, XiaMen BOSON biotech Co. Ltd, Fujian Province,China) were positive in CSF with negative culture results of bacteria and fungus. Serum Epstein-Barr virus-IgM antibody, herpes simplex virus -IgM antibody, cytomegalovirus-IgM antibody were all negative. The antiganglioside antibodies (AGA) (Beijing Hightrust Diagnostics, Co. Ltd., China) were negative in both peripheral blood and CSF. No pathogens had been detected by the metagenomic next-generation sequencing (mNGS, BGI-Shenzhen, Guangdong Province, China) using CSF sample. The electromyography examination revealed peripheral motor and sensory nerve injuries involving with demyelinating and nerve axon. The abnormal results included slowed conduction, prolonged latency and reduced amplitude of evoked velocity located in motor nerves involving with bilateral median nerve, bilateral ulnar nerve and bilateral common peroneal nerve, and slowed conduction located in sensory nerves involving with bilateral median nerve, bilateral ulnar nerve and bilateral medial plantar nerve. The slowed conduction was also observed in F waves of bilateral ulnar nerve. Hantavirus infection-associated AIDP was considered, which agreed with Brighton criteria level 1 and the criteria of National Institute of Neurological Disorder and stroke.^[22,23]^ Then the paralysis progression was observed in his double lower limbs. The proximal muscle strength of both lower limbs was grade 2 while the distal muscle strength of both lower limbs was grade 1. After IVIG (0.4 g/kg) for 5 days, the paralysis progression stopped, followed by a vanished numbness and a slow muscle power recovery. At day 14 of admission, he adopted a structured rehabilitation program.^[24,25]^ The physical therapy, occupational therapy, electric standing bed, low-frequency electrical stimulation and acupuncture were used. A month later, his limb function was significantly improved. On discharge (May 31, 2023), he could realize 50 m of walking distance on his own. On August 30, 2023, he could walk fluently without facial paralysis.

## 3. Discussion and conclusions

This case was characterized by mild HFRS as the first presentation and subsequent AIDP as the second presentation, who underwent a multidisciplinary treatment and got a rapidly clinical improvement. The clinical features from previous reported 6 GBS cases of Hantavirus infection were summarized in Figure 1. A case with Hantavirus infection-related chronic inflammatory demyelinating polyneuropathy was excluded.^[20]^ In total, 4 cases came from Europe and 2 came from Asia. They presented GBS at 1 to 2 weeks after HFRS onset. The distribution of HFRS clinical types were mild (2 cases), moderate (1 case), and severe (3 cases). There were 4 male cases and 2 female cases. The ages were between 27 and 62 years old. Albuminocytologic dissociation was found in 5 cases. The MRI examination was carried out in 2 cases. Spinal images on gadolinium-enhanced MRI in our case showed linear enhancement at the nerve root, which was identical to previous report.^[26]^ The AGA detection has not been ordinarily carried out in Hantavirus-infected cases with GBS. Nerve conduction studies can identify various subtypes, which was carried out in 5 cases. IVIG were applied to 3 patients and plasma-exchange were applied to 2 patients. There were 3 cases with respiratory distress and 2 cases required mechanical ventilation. All cases survived. A mild form of HFRS was commonly misdiagnosed at admission. According to clinical presentation, epidemiological data, and positive serological Hantavirus IgM or 4 times elevation of Hantavirus IgG using double serum samples, Hantavirus infection could be verified.^[27]^ Furthermore, reverse transcription-polymerase chain reaction assays was also an alternative diagnosis tool.^[28]^ Although the incidence of HFRS in China gradually decreased, which in Liaoning was still moderately endemic especially in spring and winter.^[29]^ As a common etiology of acute flaccid paralysis, GBS is an immune-mediated disease, which usually occurs within 2 weeks postantecedent infection.^[30]^ Although most patients recover well, GBS could present a life-threatening condition, treatment-related fluctuations/relapses and a long-term sequela. Plasma-exchange or IVIG should be early applied to treat GBS as soon as possible.

## Acknowledgments

We thank the patient for agreeing to submit this report. We thank the Wang JinYong (Department of Infectious Diseases II of the First Affiliated Hospital of China Medical University) for his professional assistance.

## Author contributions

Data curation: Ying Zhou, Jun Yang, Hong Hai, Jun Dong.

**Resources:** Ying Zhou, Jun Yang, Hong Hai.

**Writing—original draft:** Ying Zhou, Ying Wen.

**Investigation:** Hong Hai, Jun Dong.

**Supervision:** Jun Dong, Ying Wen.

**Project administration:** Ying Wen.

**Writing—review & editing:** Ying Wen.

## References

[1] Hautala N Partanen T Kubin AM. Central nervous system and ocular manifestations in puumala hantavirus infection. Viruses. 2021;13:1040. 34072819 10.3390/v13061040 PMC8229408

[2] Partanen T Chen J Lehtonen J. Heterozygous TLR3 mutation in patients with Hantavirus encephalitis. J Clin Immunol. 2020;40:1156–62. 32936395 10.1007/s10875-020-00834-2 PMC7567724

[3] Lebecque O Mulquin N Dupont M. Cytotoxic lesion of the corpus callosum caused by Puumala Hantavirus infection. J Belg Soc Radiol. 2019;103:11. 30693350 10.5334/jbsr.1616 PMC6344642

[4] Bergmann F Krone B Bleich S. Encephalitis due to a Hantavirus infection. J Infect. 2002;45:58–9. 12217734 10.1053/jinf.2002.1014

[5] Hautala T Hautala N Mähönen S-M. Young male patients are at elevated risk of developing serious central nervous system complications during Acute Puumala Hantavirus infection. BMC Infect Dis. 2011;11:217. 21838931 10.1186/1471-2334-11-217 PMC3166934

[6] Hautala T Sironen T Vapalahti O. Hypophyseal hemorrhage and panhypopituitarism during Puumala virus infection: magnetic resonance imaging and detection of viral antigen in the hypophysis. Clin Infect Dis. 2002;35:96–101. 12060884 10.1086/340859

[7] Valtonen M Kauppila M Kotilainen P. Four fatal cases of nephropathia epidemica. Scand J Infect Dis. 1995;27:515–7. 8588146 10.3109/00365549509047057

[8] Hautala T Mähönen S-M Sironen T. Central nervous system-related symptoms and findings are common in acute Puumala Hantavirus infection. Ann Med. 2010;42:344–51. 20545485 10.3109/07853890.2010.480979

[9] Forslund T Saltevo J Anttinen J. Complications of nephropathia epidemica: three cases. J Intern Med. 1992;232:87–90. 1353521 10.1111/j.1365-2796.1992.tb00555.x

[10] Xie D Xu W Xian Y. Rare case of intracranial hemorrhage associated with seoul virus infection diagnosed by metagenomic next-generation sequencing. J Clin Lab Anal. 2021;35:e23616. 33084078 10.1002/jcla.23616 PMC7891533

[11] Chen H Li Y Zhang P. A case report of empty Sella syndrome secondary to Hantaan virus infection and review of the literature. Medicine (Baltimore). 2020;99:e19734. 32243412 10.1097/MD.0000000000019734 PMC7220083

[12] Jovanović D Kovacević Z Dragović T. Anterior pituitary lobe atrophy as late complication of hemorrhagic fever with renal syndrome. Vojnosanit Pregl. 2009;66:166–8. 19281130 10.2298/vsp0902166j

[13] Ahn HJ Chung JH Kim DM. Hemorrhagic fever with renal syndrome accompanied by panhypopituitarism and central diabetes insipidus: a case report. J Neurovirol. 2018;24:382–7. 29508304 10.1007/s13365-018-0624-6

[14] Pekic S Cvijovic G Stojanovic M. Hypopituitarism as a late complication of hemorrhagic fever. Endocrine. 2005;26:79–82. 15888918 10.1385/ENDO:26:2:079

[15] Bhoelan S Langerak T Noack D. Hypopituitarism after Orthohantavirus infection: what is currently known? Viruses. 2019;11:340. 30974852 10.3390/v11040340 PMC6521286

[16] Esselink RA Gerding MN Brouwers PJ. Guillain-Barre syndrome associated with hantavirus infection. Lancet. 1994;343:180–1. 10.1016/s0140-6736(94)90975-x 7904032

[17] Rabaud C May T Hoen B. Guillain-Barre syndrome associated with hantavirus infection. Clin Infect Dis. 1995;20:477–8. 7742467 10.1093/clinids/20.2.477

[18] Tassart G Balbeur S Deltombe T. Guillain-Barre syndrome associated with Puumula hantavirus infection. Acta Clin Belg. 2014;69:371–4. 25092197 10.1179/0001551214Z.00000000085

[19] Jiao J Wu L Yin J. Guillain-Barre syndrome associated with hemorrhagic fever with renal syndrome in China: a case report. BMC Infect Dis. 2018;18:143. 29587642 10.1186/s12879-018-3049-1 PMC5872525

[20] Lim JY Lim YH Choi EH. Acute-onset chronic inflammatory demyelinating polyneuropathy in hantavirus and hepatitis B virus coinfection: a case report. Medicine (Baltimore). 2016;95:e5580. 27930572 10.1097/MD.0000000000005580 PMC5266044

[21] Lebecque O Dupont M. Puumala Hantavirus: an imaging review. Acta Radiol. 2020;61:1072–9. 31805769 10.1177/0284185119889564

[22] Asbury AK Cornblath DR. Assessment of current diagnostic criteria for Guillain-Barré syndrome. Ann Neurol. 1990;27(Suppl. 1):S21–4. 2194422 10.1002/ana.410270707

[23] Sejvar JJ Kohl KS Gidudu J.; Brighton Collaboration GBS Working Group. Guillain-Barré syndrome and Fisher syndrome: case definitions and guidelines for collection, analysis, and presentation of immunization safety data. Vaccine. 2011;29:599–612. 20600491 10.1016/j.vaccine.2010.06.003

[24] Meythaler JM. Rehabilitation of Guillain-Barré syndrome. Arch Phys Med Rehabil. 1997;78:872–9. 9344309 10.1016/s0003-9993(97)90203-3

[25] Kapre JP Harjpal P Samal SS. Early approach towards atypical Guillain-Barré syndrome: a physiotherapy perspective in a case report. Cureus. 2022;14:e31235. 36514603 10.7759/cureus.31235 PMC9733801

[26] Yikilmaz A Doganay S Gumus H. Magnetic resonance imaging of child-hood Guillain-Barre syndrome. Childs Nerv Syst. 2010;26:1103–8. 20556395 10.1007/s00381-010-1197-8

[27] Elgh F Lundkvist A Alexeyev OA. Serological diagnosis of hantavirus infections by an enzyme-linked immunosorbent assay based on detection of immunoglobulin G and M responses to recombinant nucleocapsid proteins of five viral serotypes. J Clin Microbiol. 1997;35:1122–30. 9114393 10.1128/jcm.35.5.1122-1130.1997 PMC232715

[28] Nunes BTD de Mendonça MHR Simith DB. Development of RT-qPCR and semi-nested RT-PCR assays for molecular diagnosis of hantavirus pulmonary syndrome. PLoS Negl Trop Dis. 2019;13:e0007884. 31877142 10.1371/journal.pntd.0007884 PMC6932758

[29] Wang Q Yue M Yao P. Epidemic trend and molecular evolution of HV family in the main hantavirus epidemic areas from 2004 to 2016, in P.R. China. Front Cell Infect Microbiol. 2021;10:584814. 33614521 10.3389/fcimb.2020.584814 PMC7886990

[30] Shahrizaila N Lehmann HC Kuwabara S. Guillain-Barré syndrome. Lancet. 2021;397:1214–28. 33647239 10.1016/S0140-6736(21)00517-1

